# Isolation and fine mapping of *Rps6*: an intermediate host resistance gene in barley to wheat stripe rust

**DOI:** 10.1007/s00122-015-2659-x

**Published:** 2016-01-11

**Authors:** Andrew M. Dawson, John N. Ferguson, Matthew Gardiner, Phon Green, Amelia Hubbard, Matthew J. Moscou

**Affiliations:** The Sainsbury Laboratory, Norwich Research Park, Norwich, NR4 7UH UK; School of Biological Sciences, University of Essex, Colchester, CO4 3SQ UK; National Institute of Agricultural Botany, Huntingdon Road, Cambridge, CB3 0LE UK

## Abstract

*****Key message***:**

**We uncouple host and nonhost resistance in barley to*Puccinia striiformis*ff. spp.*hordei*and*tritici*. We isolate, fine map, and physically anchor*Rps6*to chromosome 7H in barley.**

**Abstract:**

A plant may be considered a nonhost of a pathogen if all known genotypes of a plant species are resistant to all known isolates of a pathogen species. However, if a small number of genotypes are susceptible to some known isolates of a pathogen species this plant may be considered an intermediate host. Barley (*Hordeum vulgare*) is an intermediate host for *Puccinia striiformis* f. sp. *tritici* (*Pst*), the causal agent of wheat stripe rust. We wanted to understand the genetic architecture underlying resistance to *Pst* and to determine whether any overlap exists with resistance to the host pathogen, *Puccinia striiformis* f. sp. *hordei* (*Psh*). We mapped *Pst* resistance to chromosome 7H and show that host and intermediate host resistance is genetically uncoupled. Therefore, we designate this resistance locus *Rps6*. We used phenotypic and genotypic selection on F_2:3_ families to isolate *Rps6* and fine mapped the locus to a 0.1 cM region. Anchoring of the *Rps6* locus to the barley physical map placed the region on a single fingerprinted contig spanning a physical region of 267 kb. Efforts are now underway to sequence the minimal tiling path and to delimit the physical region harboring *Rps6*. This will facilitate additional marker development and permit identification of candidate genes in the region.

**Electronic supplementary material:**

The online version of this article (doi:10.1007/s00122-015-2659-x) contains supplementary material, which is available to authorized users.

## Introduction

Nonhost resistance is often described as the complete resistance of an entire plant species to a specific pathogen (Heath 2000; Mysore and Ryu 2004; Nürnberger and Lipka 2005). In the majority of cases, this definition will hold true, as generally, most plants remain healthy, despite the ubiquity of potentially pathogenic microbes in the environment. However, it is clear that some plant pathogen interactions do not prescribe to the qualitative separation of host and nonhost. Instead, they appear to exist in a transitional phase between the two states, where radial coevolution with microbial species leads to the erosion, or reinforcement, of host status to pathogenic microbes (Niks and Marcel 2009; Schulze-Lefert and Panstruga 2011). This ‘coevolution’ can be considered a short-term interaction relative to the evolutionary time of plant speciation. Under long-term timescales, the preponderance of evidence supports host-shift speciation rather than cospeciation in the evolution of plant and microbial species (de Vienne et al. 2013). In contrast, our understanding of the short-term dynamics of host specialization remains poorly understood.

Host specialization is often observed in the interaction of mildew and rust fungi with grasses, particularly the *formae speciales* divide of cereal rusts (Bushnell and Roelfs 1984; Eriksson 1894; Niks and Marcel 2009). Eriksson (1894) first proposed the *formae speciales* to differentiate forms of cereal rusts that were pathogenically specialized to given host genera but were otherwise morphologically indistinguishable. However, it was found that the *formae speciales* were not exclusively restricted to their host genera (Straib 1937) and the application to plant species outside of the host genera can result in varying degrees of compatibility: ranging from haustoria formation and hyphal colonization continuing through to lifecycle completion and pustule formation (Bettgenhaeuser et al. 2014). Despite the observation of non-exclusivity, the *formae speciales* division has been maintained. Bettgenhaeuser et al. (2014) proposed that interactions involving inappropriate *formae speciales* and nonhost plant genera are intermediate host systems that exist in the evolutionary transition between host and nonhost.

To date, a number of studies have reported on the genetic architecture of intermediate host systems with the majority reporting evidence for the role of major loci underlying resistance to nonhost *formae speciales* (Jafary et al. 2006, 2008; Pahalawatta and Chen 2005; Sui et al. 2010; Tosa 1989, 1992). So far, no major locus conditioning intermediate host resistance has been cloned within the Triticeae tribe. However, numerous major loci have been cloned for host pathosystems with the majority coding for intracellular, nucleotide-binding, leucine-rich repeat proteins (NLRs) (Krattinger et al. 2009). Whether the same observations will be made for major loci in intermediate systems is unclear. However, the proposed contribution of NLRs to nonhost immunity is now widely accepted despite the relatively few well-characterized examples (Mysore and Ryu 2004; Schulze-Lefert and Panstruga 2011; Thordal-Christensen 2003). Molecular characterization of two tandemly arranged NLRs, *RGA4* (*Resistance gene analogue 4*) and *RGA5* (*Resistance gene analogue 5*), have been shown to condition *Pi*-*CO39*(*t*) mediated resistance to a nonhost *Magnaporthe oryzae* (rice blast) isolate in rice (Cesari et al. 2013). Similarly, *WRR4* conditions nonhost resistance to *Albugo candida*, the causal agent of white blister rust, in *Arabidopsis thaliana* (Borhan et al. 2008). These observations support the molecular evolutionary model proposed by Schulze-Lefert and Panstruga (2011) that implicates NLRs in nonhost resistance. In the model, the authors assert that the contribution of NLR triggered immunity will decrease as a function of evolutionary divergence time from the host. Given the presumed evolutionary infancy of the *formae speciales* divide, one may hypothesize that major loci governing nonhost resistance in intermediate host systems may be underpinned by NLRs analogous to host systems. However, very little evidence exists to support this notion due to a lack of well-resourced, model pathosystems, with robust phenotypes, that permit the elucidation of the underlying molecular mechanisms of resistance.

Barley (*Hordeum vulgare* L.) has many traits that make it an appealing model organism. It is an inbreeding crop, a true diploid, and has a rich pedigree of genetic research that spans more than a century (Ullrich 2010). Despite its large genome of 5.1 Gbp that is largely composed of repetitive DNA, barley has been proposed as a model for genomic research within the Triticeae tribe (IBGSC 2012; Schulte et al. 2009) and to date, there have been >20 genes isolated via map-based cloning approaches (Ariyadasa et al. 2014; Krattinger et al. 2009). Recently, significant advances have been made with regards to the genetic and genomic resources available in barley and these hold significant promise to assist gene isolation studies (Mayer et al. 2011; Muñoz-Amatriaín et al. 2011). The first major step towards a draft genome sequence was made when the International Barley Genome Sequencing Consortium published a 4.98 Gbp BAC-based physical map anchored to a high-resolution genetic map (IBGSC 2012). In this study, sequencing of 6278 BAC clones and 304,523 BAC end sequences (BES) allowed 112,989 whole genome shotgun (WGS) contiguous sequences (contigs) to be anchored to the physical map. Additionally, an estimation of the gene space was made by aligning full-length barley cDNAs and over 1.5 billion RNAseq reads to the WGS assembly resulting in the identification of over 26,000 high confidence genes (IBGSC 2012). Shortly after the publication of the anchored physical map, Mascher et al. (2013) used low read depth sequencing of progeny from a recombinant inbred line (RIL) population (POPSEQ) to genetically bin approximately 1.2 Gbp of sequence information. Subsequently, the integration of these two datasets and the anchoring of additional sequence information via multiple genetic maps led to the publication of a barley genomic resource, spanning ~98 % of the barley genome, genetically anchored by two million single nucleotide polymorphisms (SNPs) (Ariyadasa et al. 2014). This resource will provide an invaluable tool for future gene isolation studies, as it provides physical sequence information that can be used for marker development, candidate gene analysis, and the generation of high confidence gene models.

In this study, we test whether there is an overlap between resistance to the host pathogen, *Puccinia striiformis* Westend. f. sp. *hordei* Erikss. (*Psh*) and the intermediate host pathogen, *Puccinia striiformis* f. sp. *tritici* Erikss. (*Pst*). We use the barley accession Abed Binder 12 that contains the *Psh* resistance gene *rps2* (Nover and Scholz 1969), which is also highly resistant to *Pst*. After mapping *Pst* resistance to chromosome 7H, we determine that host and intermediate host resistance are uncoupled and designate the *Pst* resistance locus *Rps6*. Subsequently, we isolate and fine map *Rps6* to a 0.1 cM region and anchor the region to a single fingerprinted contig (FPC) in barley. Future work on the cloning of *Rps6* will establish the genetic basis for resistance and its contribution to host and nonhost resistance.

## Materials and methods

### Plant materials

Seed for Abed Binder 12 (PI 327961) and Russell (PI 483127) were obtained from the United States Department of Agriculture, Agricultural Research Service (USDA-ARS). A cross was made using Abed Binder 12 as the maternal parent and Russell as the paternal pollen donor. A single F_1_ plant was used to generate three independent F_2_ populations used for inoculation with *Psh* and *Pst*, and for the development of a F_2:3_ population.

### Pathogen materials and assays

Pathogen assays were carried out using either *Pst* isolates 08/501 or 08/21, or *Psh* isolate B01/2. The *Psh* and *Pst* isolates were collected by The National Institute for Agricultural Botany in 2001 and 2008, respectively. *Pst* isolates 08/21 and 08/501 urediniospores were bulked, and maintained, on the susceptible wheat cvs. Solstice and Victo, respectively. *Psh* isolate B01/2 urediniospores were bulked, and maintained, on the susceptible barley cv. Cassata. For plant inoculations, four groups of eight seeds were sown in a 1 L pot using a peat-based compost. Plants were grown in a controlled environment chamber at 18 °C day and 11 °C night using a 16 h light and 8 h dark cycle with lighting provided by metal halide bulbs (Philips MASTER HPI-T Plus 400 W/645 E40). Inoculations were performed on 14-day-old seedlings when the first leaf was fully emerged and prior to the emergence of the second leaf. Inoculum was prepared by mixing fresh spores with talcum powder at a weight ratio of 1:16. A compressed air pump was used to disseminate inoculum onto seedlings positioned on a spinning platform. After inoculation, seedling pots were sealed in plastic bags and stored in the dark at 6 °C to achieve the high humidity required for successful germination. Seedlings were returned to the controlled environment growth chamber after 48–72 h post inoculation. Disease symptoms were scored 14 days post inoculation.

### Macroscopic phenotyping

Plants inoculated with *Psh* were phenotyped macroscopically using the McNeal scale: a scale designed for host systems that ranges from 0 (immune; no visible symptoms) to 9 (very susceptible; abundant pustule formation, without chlorosis) (McNeal et al. 1971). For plants inoculated with *Pst*, we used an alternate phenotyping scale to measure the macroscopic phenotypes of chlorosis (leaf yellowing) and infection (pustule formation). They were individually scored on a continuous nine-point scale ranging from 0 to 4, with increments of 0.5. Scores reflected the percentage of the inoculated leaf surface expressing the disease symptom. A score of 0 indicated no expression of the phenotype (0 % coverage), whereas a score of 4 indicated extensive expression of the phenotype (100 % coverage).

### Microscopic phenotyping

First leaves of inoculated seedlings were harvested with scissors and placed in 15 mL centrifuge tubes filled with 1.0 M KOH and a droplet of surfactant (Silwet L-77, Loveland Industries Ltd.). Tubes were incubated at 37 °C for 12–16 h. The KOH solution was decanted and leaves were washed three times using 15 mL of 50 mM Tris HCL-pH 7.5. Leaf samples were then incubated overnight at 4 °C in a 2.0 % w/v staining solution containing wheat germ agglutinin conjugated to fluorescein isothiocyanate (WGA-FITC; Sigma Aldrich; L4895-10MG) dissolved in 50 mM Tris HCL. Leaves were washed with sterile water and mounted on microscope slides. Mounts were visualized under blue light excitation using a fluorescence microscope with GFP filter under a 5× objective. Each field of view (FOV) was 2.72 mm × 2.04 mm. Data was collected by estimating the amount of colonization and pustule formation in non-overlapping FOVs covering the length and breadth of the leaf. Disease symptoms were estimated to be less than 15 %, between 15 and 50 %, or greater than 50 % by assigning the values 0, 0.5 and 1 to each FOV. Percent colonization (pCOL) and pustule formation (pPUST) scores, ranging from 0 to 100 %, were calculated by averaging the values relative to the number of FOVs in each leaf.

### DNA extraction

DNA from all populations was extracted from leaf tissue following a CTAB-based protocol adapted for 96-well based format modified from (Stewart and Via 1993) that provides PCR-grade genomic DNA (Nick Lauter, personal communication).

### Marker development for genetic map construction

The concentration of gDNA was estimated using the PicoGreen dsDNA quantification assay (Life Technologies; P11496) and was normalized to 60 ng/μL. Oligonucleotide assay (OPA) genotyping using the barley BOPA1 design that includes 1536 SNP-based markers was performed at the University of California, Los Angeles Southern California Genotyping Consortium (Los Angeles, CA, USA) (Close et al. 2009). Additional markers were developed as either cleaved amplified polymorphic sequence (CAPS) or Sequenom MassARRAY markers to bridge gaps between unlinked chromosome arms and increase marker density. For CAPS marker development, we identified type II restriction enzymes that digest at polymorphic positions using CAPS Designer (http://solgenomics.net/tools/caps_designer/caps_input.pl). CAPS marker PCR reactions were prepared by mixing 2 μL buffer (10×), 0.4 μL dNTPs, 0.4 μL forward primer, 0.4 μL reverse primer, 0.2 μL Taq polymerase, 2 μL gDNA at 10 ng/μL, and 14.6 μL H_2_O. The PCR cycling started with an initial denaturation step at 94 °C for 5 min and then proceeded through a cycle of 94 °C for 20 s, annealing at 56 °C for 30 s and primer extension at 72 °C for 1 min for a total of 35 cycles. The procedure ended with a final extension at 72 °C for 5 min before being held at 16 °C. Digestions were performed according to the manufacturer’s instructions for individual enzymes. Electrophoresis was used to resolve restriction fragments using 2.0 % TBE agarose gels stained with ethidium bromide. Gel images were taken using a Bio-Rad ChemDoc XRS + imaging system and markers were scored manually. GBS CAPS markers are described in (Kota et al. 2008). All primers and restriction enzymes for CAPS markers are detailed in ESM 1. For Sequenom marker development, SNP sequences were extracted in IUPAC format with 40–60 bp flanking sequence. This sequence was used as a template for primer design using MassARRAY software v3.1 for the multiplexing up to 32 SNP assays. Sequenom genotyping was carried out at the Iowa State University Genomic Technologies Facility (Ames, IA, USA). All SNPs and WGS contig source information for Sequenom markers are detailed in ESM 2.

### Genetic map construction

A genetic map was constructed using 589 markers including 535 barley OPA (Close et al. 2009), 26 CAPS markers, and 28 Sequenom markers. JoinMap v4 was used using default parameters and an independence LOD threshold of 4.0 (van Ooijen 2006). Genetic distances were estimated using the Kosambi mapping function. Integrity of the genetic map was evaluated through comparison with the current OPA consensus genetic map of barley (Muñoz-Amatriaín et al. 2011) and with two-point linkage tests using R/qtl (v1.33-7).

### QTL and ANOVA analyses

Composite interval mapping was performed with QTL Cartographer (v1.17j) using model 6, the selection of five background markers, a step size of 2 cM, and a window size of 10 cM (Basten et al. 1994). Significant QTLs were extracted using the Eqtl module under the H_0_:H_3_ model using experiment-wide thresholds (EWT) that were calculated using 1000 permutations with the reselection of background markers using a threshold of *α* < 0.05 (Doerge and Churchill 1996; Lauter et al. 2008). ANOVA analyses for testing the linkage of individual markers were performed with R/qtl.

### Transcriptome sequencing and assembly

Leaf tissue was harvested from first and second leaves 18 days after sowing for Abed Binder 12 and Russell. Samples were flash frozen in liquid nitrogen, and stored at −80 °C. Samples were homogenized in liquid nitrogen-chilled pestle and mortars. RNA was extracted from samples using TRI-reagent (Sigma-Aldrich; T9424) according to the manufacturer’s protocol. DNA was removed by treating samples with RQ1 RNase free DNase (Promega; M6101). Samples were purified using RNeasy mini spin columns following the RNA Cleanup protocol (Qiagen; product No. 74104). The quality and integrity of the RNA samples were assessed using RNA Nano Chips (Agilent Technologies; product no. 5067-1511) on an Agilent 2100 Bioanalyzer. Abed Binder 12 and Russell RNA libraries were constructed using Illumina TruSeq RNA library preparation (Illumina; RS-122-2001). Final library insert sizes were predicted to be 411 and 339 bp for Abed Binder 12 and Russell, respectively. Barcoded libraries were sequenced using 100 bp paired-end reads on one lane of a Hiseq 2000/2500. This generated 32.0 and 59.3 million paired end reads for Abed Binder 12 and Russell, respectively. All library preparation and sequencing was performed at The Genome Analysis Centre (Norwich, UK). RNAseq data quality was assessed with FastQC and reads were removed using Trimmomatic (v0.32) with parameters set at ILLUMINACLIP:TruSeq 3-PE.fa:2:30:10, LEADING:3, TRAILING:3, SLIDINGWINDOW:4:15, and MINLEN:100. These parameters will remove all reads with adapter sequence, ambiguous bases, or a substantial reduction in read quality. Transcriptome assembly was performed using Trinity (v2013-11-10) using default parameters. Raw reads have been submitted to NCBI Short Read Archive under the BioProject ID PRJNA292371 and SRA accession SRR2153288 (cv. Abed Binder 12) and SRR2153285 (cv. Russell).

### Marker development for saturation at the *Rps6* locus

Initial marker development was guided by two approaches to identify sequences anchored to the *Rps6* region. This included the identification of anchored unigenes based on marker colinearity with existing genetic maps (Moscou et al. 2011; Muñoz-Amatriaín et al. 2011; Potokina et al. 2008) and orthologous rice genes based on the barley genome zipper (Mayer et al. 2011). A region on rice chromosome 6 was selected including 38 genes (Os06g43140 to Os06g43900). Best BLASTn hits returned from the cv. Morex WGS assembly (IBGSC 2012) were used as template for PCR primer design using Primer3 (libprimer3 release 2.3.6). All BLASTn queries were performed using blastall (v2.2.23). Abed Binder 12 and Russell gDNA was used as template for PCR amplification and Sanger sequencing. SNPs were identified by aligning sequence files using Seqman software (DNAstar Lasergene v11). SNPs were then used to develop markers using Cleaved Amplified Polymorphic Sequences or Sequenom MassARRAY iPLEX platform as described above.

Subsequent marker development involved either (1) the comparison of genomic contigs derived from cvs. Barke, Bowman, and Morex or (2) the comparison of Abed Binder 12 and Russell RNAseq aligned reads to WGS contigs anchored to the *Rps6* region (IBGSC 2012; Mascher et al. 2013). Geneious (v8.1.6) was used for read alignment using Geneious mapping function with default parameters and data visualization (Kearse et al. 2012). SNPs were converted into Kompetitive Allele Specific PCR (KASP) markers using a similar approach as described in (Ramirez-Gonzalez et al. 2015). All WGS contig source information, SNPs, KASP marker template, and primers are detailed in ESM 3. KASP assays were performed at the John Innes Centre Genotyping Facility (Norwich, UK).

### Recombination screen and phenotyping

A recombination screen was carried out using seed bulked from F_3_ plants selected from a single F_2:3_ family that were heterozygous for *Rps6*. Sequenom markers were converted into KASP markers and used as flanking markers to identify recombinant chromosomes. Two independent progeny tests were performed using individuals with recombinant chromosomes. A total of 16 individuals per family per replicate were scored for macroscopic observation of chlorosis and infection.

## Results

**Fig. 1:**
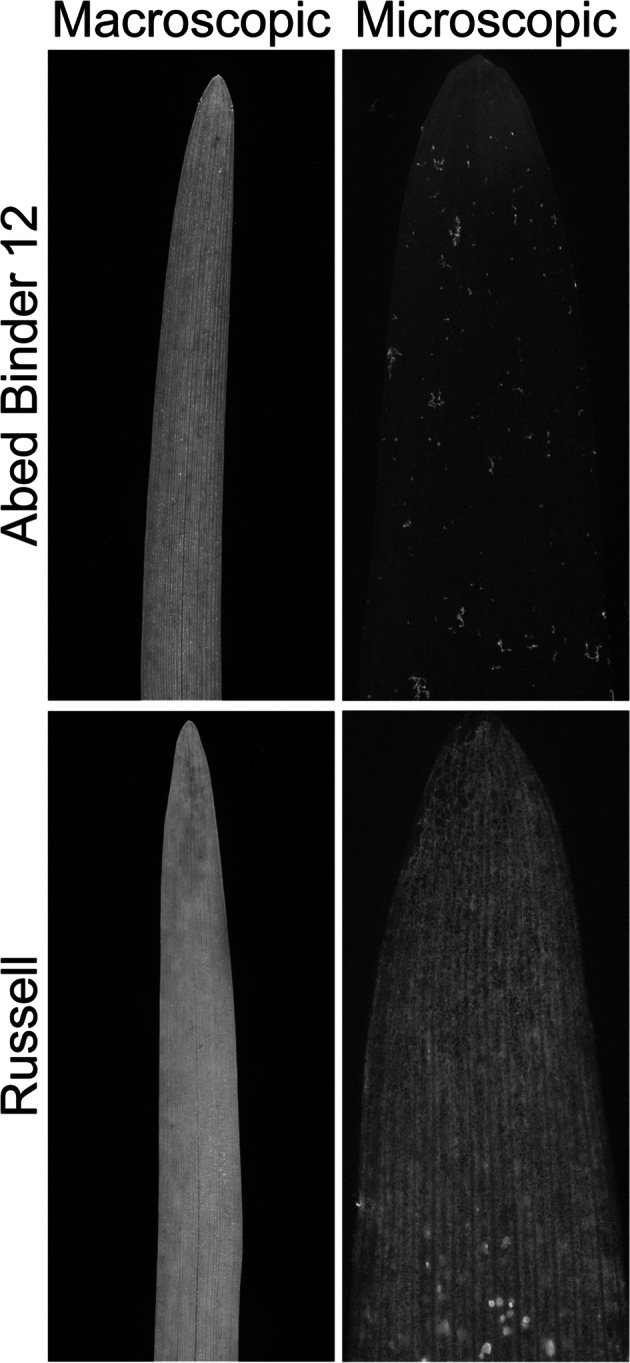
Macroscopic and microscopic phenotypes of cultivars Abed Binder 12 and Russell inoculated with *Pst*

**Fig. 2:**
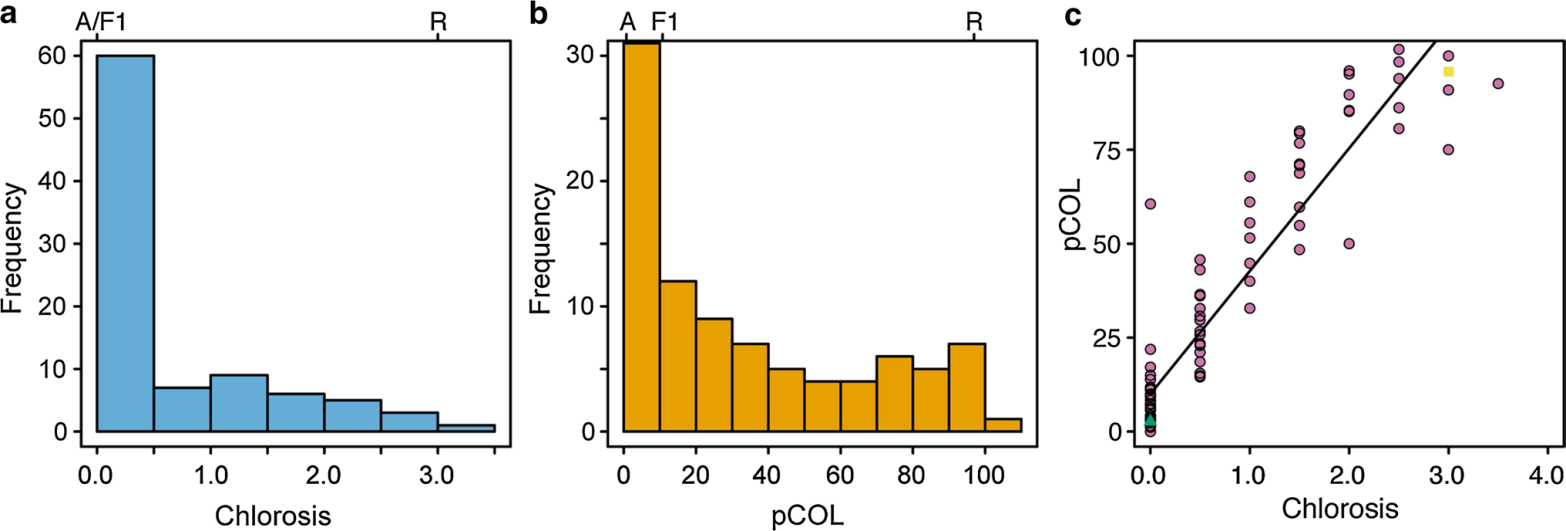
Histograms and two-way plot of chlorosis and colonization on the Abed Binder 12 × Russell F_2_ population inoculated with *Pst* isolate 08/501. Histograms showing the segregation of chlorosis (**a**) and pCOL (**b**) in the F_2_ population. Parental and F_1_ phenotypes shown above plots (*A* Abed Binder 12, *R* Russell). **c** Two-way plot showing correlation of chlorosis and pCOL phenotypes. The phenotypes of Abed Binder 12 and Russell are shown as the *green triangle* and *yellow square*, respectively

Our initial hypothesis was that resistance to host pathogens would overlap with resistance to intermediate host pathogens. To test this hypothesis, we focused our attention on the unmapped *Psh* resistance gene *rps2* that is present in the barley cultivar Abed Binder 12 (Nover and Scholz 1969). Screening of Abed Binder 12 found it was highly resistant (McNeal score 1) to *Psh* isolate B01/2, whereas cultivar Russell was highly susceptible (McNeal score of 8). Similar differential phenotypes were observed after inoculating Abed Binder 12 and Russell with *Pst* isolates 08/501 and 08/21, although Russell rarely showed pustules but had a clear microscopic phenotype of colonization (Fig. 1). We wanted to understand the genetic architecture of *Pst* resistance within Abed Binder 12 and to determine whether *rps2* contributes to resistance. We independently inoculated two Abed Binder 12 × Russell F_2_ populations with *Psh* isolate B01/2 (AxR-*Psh*) and *Pst* isolate 08/501 (AxR-*Pst*). In both experiments, the parents, F_1_ and 92 F_2_ plants were phenotyped using macroscopic phenotyping, and in the case of *Pst*, the microscopic evaluation of *Pst* colonization (pCOL). In the AxR-*Psh*, F_1_ and segregation of F_2_ individuals suggested the presence of a single recessive resistance gene conditioning pustule formation (28 resistant: 65 susceptible, model 1:3; *χ*^2^ = 1.29, *p* = 0.26; ESM 4). Pustule formation was not observed for the AxR-*Pst* F_2_ population, although segregation was observed for chlorosis and pCOL (Fig. 2a, b). A strong correlation between chlorosis and pCOL was observed (*r*^2^ = 0.88) (Fig. 2c). The F_1_ displayed similar resistant phenotype to Abed Binder 12, although it is difficult to ascertain the mode of inheritance without understanding the number of loci contributing to resistance.

To map resistance to *Pst*, we genotyped the AxR-*Pst* F_2_ population with the barley oligonucleotide assay (BOPA1), which interrogates 1536 SNP-based markers (Close et al. 2009). A total of 535 polymorphic OPA markers were identified between Abed Binder 12 and Russell and they were used to generate a genetic map with eight linkage groups. Chromosome 7H was the only chromosome that spanned two linkage groups. A total of 26 CAPS markers and 28 Sequenom MassARRAY markers were used to bridge gaps between unlinked chromosome arms and increase marker density. The final map consists of 589 markers over seven linkage groups, representing 362 non-redundant marker haplotypes and a total genetic distance of 1131 cM (ESM 5). On average, each non-redundant marker was separated by approximately six recombination events that equated to a mean distance of 3.1 cM. Only 21 regions had genetic distances greater than 10 cM and the greatest distance was 27.9 cM. The quality of the genetic map was assessed using two point linkage tests between markers (ESM 6). The majority of the genetic map did not exhibit segregation distortion, with only a slight reduction in heterozygosity on the long arm of chromosome 2H (marker 1_0214; *χ*^2^ = 9.65, *p* = 0.003).

**Fig. 3:**
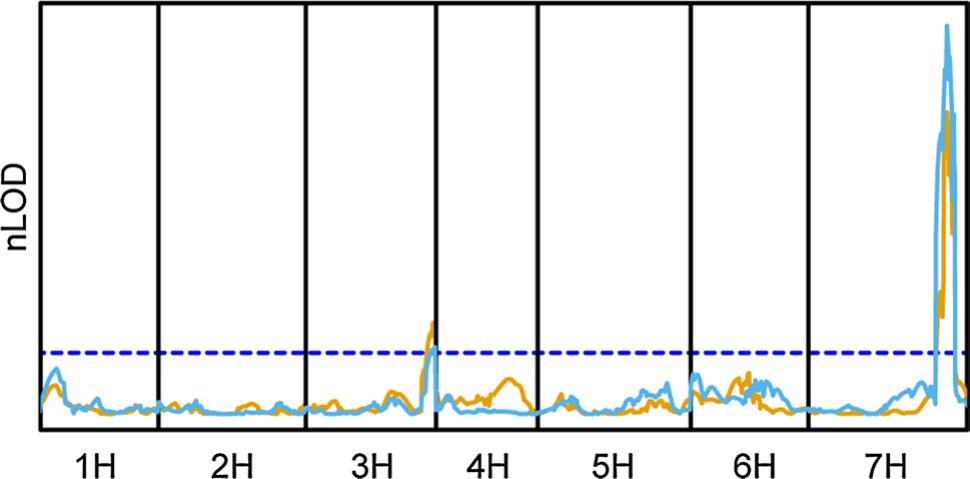
Composite interval mapping of chlorosis and pCOL phenotypes in the Abed Binder 12 × Russell F_2_ population inoculated with *Pst*. LOD curves were normalized (nLOD) for chlorosis (*sky blue*) and pCOL (*orange*) based on individual experiment-wide thresholds (*dark blue dashed line*) based on 1000 permutations. A step size of 2 cM was used, with the *x*-axis spanning the length of the AxR-*Pst* F_2_ population genetic map

We performed quantitative trait locus (QTL) analysis using composite interval mapping with chlorosis and pCOL phenotypes on the AxR-*Pst* population. We identified a major effect locus on the long arm of chromosome 7H that was contributed by Abed Binder 12 (Fig. 3). The QTL accounted for 57.7 and 69.4 % of the phenotypic variation for chlorosis and pCOL, respectively. In both instances, marker U32_7356_p1, positioned at 169.7 cM, was the most strongly linked marker. Phenotype by genotype plots using this marker showed better clustering of the susceptible lines using pCOL than chlorosis (ESM 7ab). However, despite these differences, the one and two LOD confidence intervals were consistent between the two datasets (Table 1). A second minor effect QTL was identified on chromosome 3H that explains 13.3 and 7.7 % of the phenotypic variation for chlorosis and pCOL, respectively. Interestingly, the chromosome 3H QTL is contributed by Russell. A multiple QTL model was used to test for epistasis between the QTLs on chromosomes 3H and 7H, but no significant interactions could be observed. The observation of a single major effect locus in Abed Binder 12 conditioning resistance to *Pst* prompted us to investigate potential linkage with resistance to *Psh*. We tested the SNP marker 2_0962 near the peak of the chromosome 7H QTL on both the AxR-*Pst* and AxR-*Psh* F_2_ populations. Strong linkage was observed in the AxR-*Pst* F_2_ population, whereas no linkage was observed on the AxR-*Psh* F_2_ population (ESM 8). Uncoupling of resistance to *Psh* and *Pst* indicates that the chromosome 7H locus is not *rps2*; therefore we designate this locus *Rps6*.

**Table 1:** Significant QTLs from composite interval mapping of chlorosis and pCOL phenotypes in the Abed Binder 12 × Russell F_2_ population inoculated with *Pst* isolate 08/501

Trait	Chr^a^	cM	Peak marker	EWT^b^	LOD	AEE^c^	DEE^d^	D/A^e^	PVE^f^
Chlorosis	3H	155.7	1_0893	4.38	6.52	−0.47	−0.07	0.15	0.13
Chlorosis	7H	169.7	U32_7356_p1	4.38	21.57	0.99	−0.51	−0.52	0.58
pCOL	3H	158.3	1_0694	4.20	4.60	−0.12	0.03	−0.21	0.08
pCOL	7H	169.7	U32_7356_p1	4.20	26.59	0.40	−0.15	−0.39	0.69

^a^Chromosome

^b^Experimental-wide threshold

^c^Additive effect estimate, positive values indicate the contribution of resistance from Abed Binder 12

^d^Dominance effect estimate

^e^Estimate of dominance to additivity ratio

^f^Percent of the phenotypic variation explained

The presence of a minor effect QTL in the AxR-*Pst* F_2_ population necessitated additional selection to isolate *Rps6*. We used a combination of phenotypic and genotypic selection on a second AxR F_2_ population. The F_2_ population (*N* = 96) was genotyped using markers flanking *Rps6* and the minor effect QTL on chromosome 3H. Subsequently, eight plants from every F_2:3_ family were macroscopically phenotyped using *Pst* isolate 08/21. Similar significance and effect sizes were observed for *Rps6* and the chromosome 3H QTL (ESM 9). A single F_2:3_ family was selected that was heterozygous for *Rps6*, absent for the chromosome 3H QTL, and showed clear macroscopic segregation for resistance. In an initial screen, 96 F_2:3_ plants derived from this family were inoculated with *Pst* isolate 08/21, genotyped with markers flanking *Rps6*, and phenotyped for chlorosis and pCOL. Distinct clustering was observed for *Rps6* with the marker U32_4671_p1 in contrast to the overlapping clustering within the original AxR-*Pst* F_2_ population (ESM 7). *Rps6* is additive in its contribution to chlorosis and pCOL, however, transgressive segregation was found within this selected F_2:3_ family for pustule formation. *Rps6* is dominant for conditioning resistance to pustule formation, suggesting that in a fully susceptible background it would be considered dominant.

**Fig. 4:**
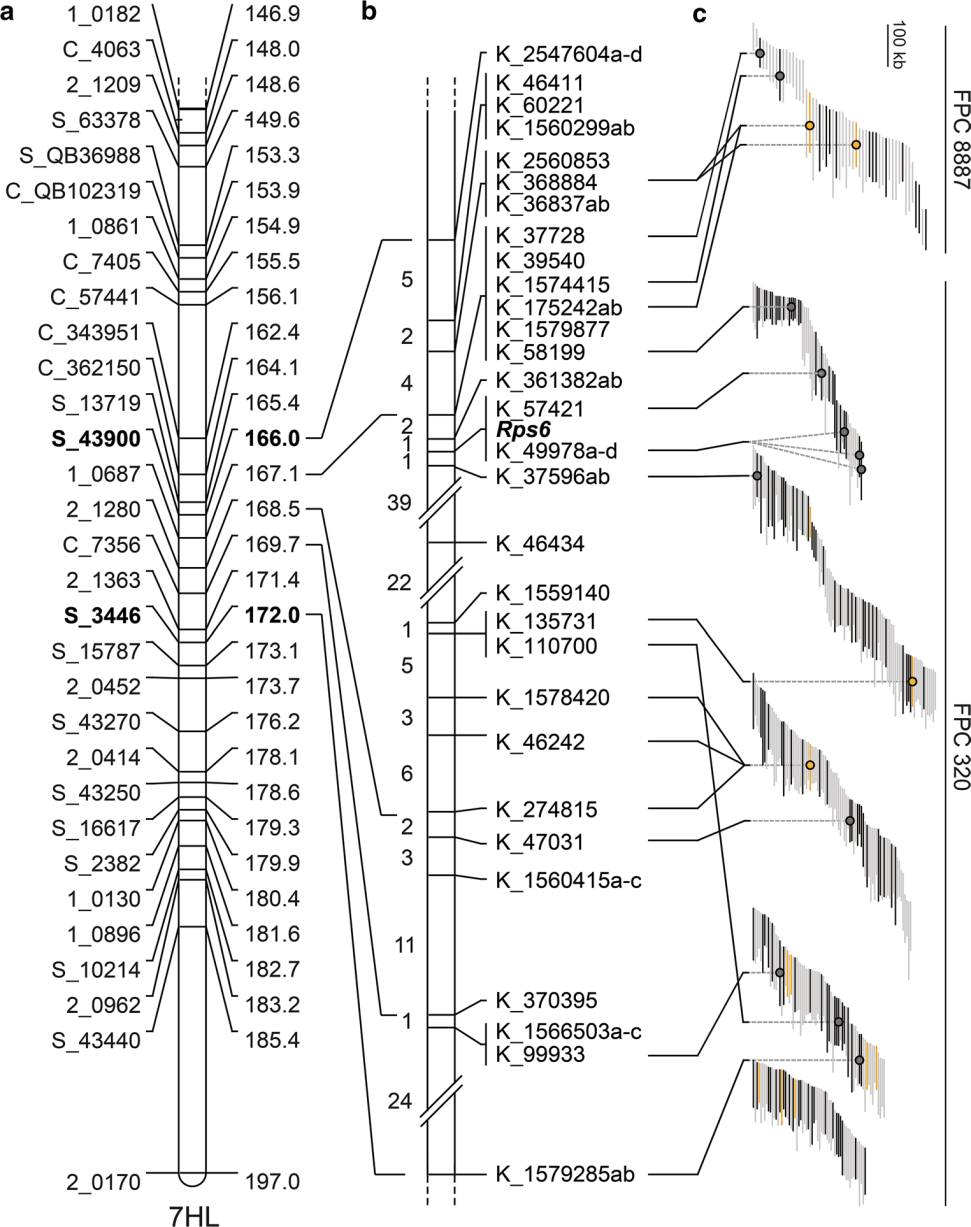
Fine mapping of *Rps6*. **a** The distal end of the long arm of chromosome 7H based on non-redundant markers harboring *Rps6* in the Abed Binder 12 × Russell F_2_ population. Sequenom markers S_43900 and S_3446 were converted into KASP markers K_2547604b and K_1579285b and were used as flanking markers for the recombination screen. **b** High-resolution genetic map based on a recombination screen including 2894 gametes. *Numbers* shown on *left* are the number of recombination events between markers. Marker names are shown on the *right*, with *letters* after marker names indicating cosegregating KASP markers derived from a single WGS contig. **c** Physical map anchoring based on the high-resolution genetic map. BACs that are sequenced or have BES available are *orange* or *black*, respectively, otherwise BACs are shown in *grey*. A truncated FPC 320 is shown based on the anchoring of markers

**Fig. 5:**
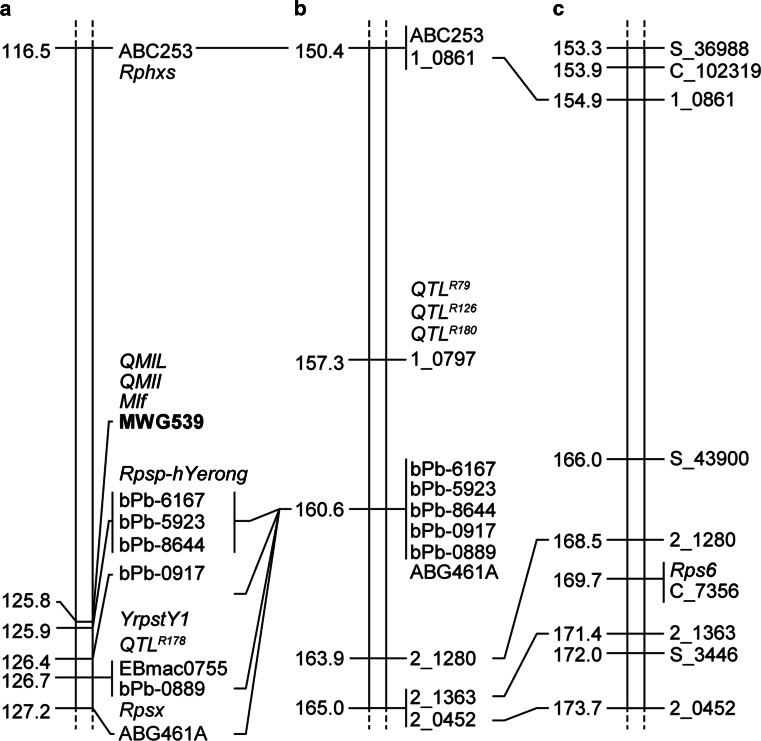
Known resistance loci in the *Rps6* region. **a** Resistance genes *Rphx*_*S*_ (Toojinda et al. 2000), *Mlf* (Schönfeld et al. 1996), *QMl*-*7H* (Backes et al. 2003), *YrpstY1* (Sui et al. 2010), *QTL* ^*R178*^ (Silvar et al. 2012), *Rpsx* (Castro et al. 2003) were mapped to the consensus map generated by Aghnoum et al. 2010. **b** Mildew resistance QTLs *QTL* ^*R79*^, *QTL* ^*R126*^, and *QTL* ^*R180*^ (Silvar et al. 2012) were mapped to the consensus map generated by (Szűcs et al. 2009). **c** Mapping of *Rps6* in the AxR-*Pst* F_2_ population. Anchoring of genes is based on the closest linked marker shown in the same *color*

To fine map *Rps6*, we carried out a recombination screen and saturated the locus with markers based on the genomic resources available in barley. The recombination screen was carried out using seed bulked from F_3_ plants that were heterozygous for *Rps6* in the previously characterized F_2:3_ family. The KASP markers K_2547604b and K_1579285b were generated from Sequenom markers S_43900 and S_3446, respectively, and used as flanking markers that span a 6.0 cM region encompassing *Rps6* (Fig. 4a). In total, 2894 gametes were characterized, identifying 135 recombination events between the flanking markers (Fig. 4b). Progeny tests were performed using individuals with recombinant chromosomes and scored homozygous or segregating for resistance, or homozygous susceptible. Additional marker saturation was required to resolve *Rps6*, so we adopted two strategies for the development of markers. In the first instance, we compared genomic contigs derived from cultivars Barke, Bowman, and Morex to identify SNPs. In parallel, we performed RNAseq on Abed Binder 12 and Russell and aligned reads to whole genome shotgun (WGS) contigs anchored to the *Rps6* region (IBGSC 2012; Mascher et al. 2013) (Fig. 4b). These analyses were performed twice; initially using the anchored contigs from the IBGSC reference anchoring that included 78 contigs between 127.12 and 129.21 cM (IBGSC 2012). Later, a larger interval was investigated including 1345 contigs between 126.20 and 131.44 cM based on an updated anchoring (Mascher et al. 2013). RNAseq data was aligned to WGS contigs and manually curated to identify SNPs polymorphic between Abed Binder 12 and Russell. A total of 102 SNPs were successfully converted into Kompetitive Allele Specific PCR (KASP) markers and surveyed on recombinant individuals in the *Rps6* region. In total, 49 KASP markers representing 30 WGS contigs mapped between the *Rps6* flanking markers (Fig. 5b). At a fine scale, contigs mapped in a different order relative to their current anchoring in the barley POPSEQ anchored contigs, although at the rough scale the general order was preserved. The markers collapsed into 18 marker bins and positioned *Rps6* in a 0.1 cM region, flanked by K_361382 (proximal) and K_37596 (distal) (Fig. 4b). *Rps6* has been resolved with maximum resolution in this recombination screen, as the gene is flanked by single recombination events with proximal and distal markers.

To anchor the *Rps6* locus to the barley physical map, we used the available BES and shotgun sequenced BACs in the *Rps6* region (IBGSC 2012). In the proximal region, several KASP markers map to the physical map on FPC 8887 based on BES and sequenced BACs. Using currently available information it is unclear if FPC 8887 is correctly orientated based on our marker order. Marker K_58199 defines a boundary on FPC 320, indicating that K_361382 is located on the physical sequence between K_58199 and K_57421. *Rps6* cosegregates with markers K_57421 and K_49978, which both map to proximal region of FPC 320. The entire distal region from K_58199 to K_1579285 is well anchored to FPC 320. Unequal rate of recombination were observed based on the physical map of barley, with extremely high rates of recombination observed between markers K_37596 and K_135731 (0.15 Mb/cM), whereas substantially lower rates of recombination were observed between markers K_58199 and K_37596 (2.58 Mb/cM). Annotated genes in the region include MLOC_18254 on contig 1579877 and two NLRs present on contigs 49978 and 37596. The high confidence gene model MLOC_65262 is present on contig 49978 and cosegregates with *Rps6* based on the resolution of our recombination screen, whereas the NLR on contig 37596 is separated by a critical recombination event. MLOC_65262 is preferentially expressed in roots, with little or no expression in leaves in Morex (IBGSC 2012). BAC sequencing along the minimal tiling path of FPC 320 will be critical for delimiting the genetic and physical interval harboring *Rps6*, in addition to permitting the full annotation of the gene content in the region.

## Discussion

In this study, we used barley as a model system for elucidating the genetic architecture determining specificity in the interaction with two *formae speciales* of *P. striiformis*. Host and intermediate host resistance were found to be uncoupled, and we identified *Rps6*, an intermediate host resistance gene in barley to *Pst*. By using phenotypic and genotypic selection on F_2:3_ families, we isolated *Rps6* for fine mapping and delimited the locus to a 0.1 cM genetic interval that encompasses approximately 267 kb.

Several resistance specificities to *Pst* have been mapped to the distal region on the long arm of chromosome 7H. Using the consensus maps that integrate multiple genotyping platforms developed by Aghnoum et al. (2010), Szűcs et al. (2009), and Muñoz-Amatriaín et al. (2011), we inferred the position of previously mapped genes (Fig. 5). We found that *Rps6* colocalizes with *YrpstY1*, a gene that confers resistance to a Chinese isolate of *Pst* in barley (Sui et al. 2010). Mapping of *YrpstY1* was achieved using nine simple sequence repeat (SSR) markers and delimited the *YrpstY1* locus to a region spans 40 cM. *Rps6* and *YrpstY1* colocalize based on the position of *EBmac0755*, the most closely linked marker to *YrpstY1*, relative to the position of *Rps6* in the AxR-*Pst* F_2_ map (Fig. 5). In parallel with our own work, *Rps6* has been independently identified and found to provide resistance in barley to *Pst* isolates from the US (Li et al. 2015). Taken together, these observations suggest that this locus is an integral component of resistance in barley to *Pst* in distinct regions around the world.

In addition to resistance to *Pst*, several resistance specificities to host and nonhost pathogens have been mapped near *Rps6* (Fig. 5). Adult plant resistance to the host pathogen *Psh* has been mapped to the *Rps6* region (Castro et al. 2003). Castro et al. (2003) identified *Rpsx* using restriction fragment length polymorphism (RFLP) markers. RFLP marker ABG461A was the closest linked marker to *Rpsx* and maps in close proximity to *Rps6* based on marker colinearity between maps (Fig. 5). Similarly, *Rphx*_*S*_, an adult plant resistance specificity to *Puccinia hordei* also mapped to the *Rps6* region on chromosome 7HL (Toojinda et al. 2000). This locus is distal to RFLP marker ABC253 and accounted for 84 % of the phenotypic variance. Derevnina et al. (2015) mapped *Rpsp*-*hYerong*, a QTL conferring resistance to *P. striiformis* f. sp. *pseudo*-*hordei* (barley grass stripe rust; BGYR), in the vicinity of *Rps6* (Derevnina et al. 2015). The DArT marker bPb-6167 was the marker underlying the peak of *Rpsp*-*hYerong* (Fig. 5). BGYR is a contemporary *formae speciales* of *P. striiformis* (Wellings et al. 2000). It is an adapted pathogen of wild *Hordeum* spp. (barley grass) and as such its interaction with barley can be considered an intermediate host pathosystem according to terminology proposed by Bettgenhaeuser et al. (2014). The observation of nonhost resistance specificities in this region also coincides with barley powdery mildew resistance including the resistance gene *Mlf* (*Mildew resistance locus f*) and several QTLs (Backes et al. 2003; Schönfeld et al. 1996; Silvar et al. 2012). The association of resistance at the *Rps6* locus to multiple diseases extends to *Magnaporthe oryzae*, wherein a minor effect QTL maps to the region (Inukai et al. 2006). It is unclear whether these specificities are due to linkage rather than pleiotropy based on current map positions as the large mapping intervals observed in most of the studies hinders our ability to draw conclusions from this data. Additional fine mapping and cloning of the genes underlying resistance will be required to conclusively define whether colocalization of these loci are due to genetic linkage or pleiotropy.

Gene nomenclature in barley requires a three-letter symbol followed by a unique number to designate the locus and a unique number or letter to define the allele (Lundqvist et al. 1997). As the nature of the *formae speciales* divide for *P. striiformis* is unclear, it is proposed to use *Rps* to identify resistance to *P. striiformis* f. sp. *tritici* (Jerome Franckowiak, personal communication). *Rps6.i* is proposed as the allele symbol for resistance contributed in Abed Binder 12 to *Pst* at the *Rps6* locus. Aside from *Psh*, *Pst* isolate specificity is currently unknown for *Rps6*, therefore either (1) the generation of near-isogenic lines or (2) gene isolation and characterization in resistant germplasm will elucidate the whether this gene has two alleles [i.e. resistant and susceptible such as *Rpg1*; (Brueggeman et al. 2002)] or multiple alleles with varying degrees of recognition specificity [such as *Mla*; (Seeholzer et al. 2010)]. Identification of transformable *Pst* susceptible barley accessions will be a critical priority and will be aided by the recently developed SusPtrit × Golden Promise doubled-haploid population (Yeo et al. 2014). SusPtrit has been shown to be susceptible to several host and nonhost pathogens of barley, including *Psh*, *Pst*, *P. striiformis* f. sp. *bromi*, and several other nonhost rust fungi (Jafary et al. 2006; Niks et al. 2013), whereas the two-row elite malting cultivar Golden Promise is well known for its ability to be transformed using *Agrobacterium tumefaciens* (Lü et al. 2015). Thus, it should be possible to select accessions that will maintain the susceptibility to *Pst* and the transformability of Golden Promise.

The success of map-based cloning is determined by the chromosomal location and physical structure of the region encompassing the gene of interest. In barley, as with many other plants, recombination rates vary along the length of the chromosome and significantly reduced rates of recombination can be observed in pericentromeric regions when compared to distal regions (IBGSC 2012). Recombination is essential for map-based gene isolation as it influences the degree to which the locus can be delimited using recombination breakpoints and the ratio of physical to genetic distance in the region. This was highlighted during the anchoring of the barley BAC-based physical map when it was estimated that the ratio of physical to genetic distance in pericentromeric regions was 10–500 times greater than in distal regions (IBGSC 2012). In the case of *Rps6*, the chromosomal localization was favorable for mapping due to its distal location on the long arm of chromosome 7H. Indeed, we observed recombination that was sufficient to delimit *Rps6* to a 0.1 cM region. Based on our current markers, we have been able to anchor *Rps6* to FPC 320 (IBGSC 2012).

The BACs spanning the *Rps6* interval, K_361382 to K_37596, have not been sequenced, but signatures of NLRs exist in the region. A critical recombinant suggests that the NLR present on contig 37596 is not *Rps6*, whereas the NLR (MLOC_65262) on contig 49978 cosegregating with *Rps6* has 16 non-synonymous differences between Abed Binder 12 and Russell (data not shown). In the parallel, fine-mapping of *Rps6* by Li et al. (2015) found resistance uncoupled from MLOC_65262, with the identification of several recombinants. NLR loci are known to be highly complex with multiple paralogs that can vary between allele based on copy number and sequence variation (Michelmore and Meyers 1998). At this time it is difficult to comprehensively identify all candidate genes in the region due to the lack of sequence information in the region. Efforts are underway to sequence the minimal tiling path of barley in Morex; this will aid additional marker development for the closing of the physical interval harboring *Rps6* and the identification of candidate genes in the region. Future work will include the development of an Abed Binder 12 BAC library and haplotype analysis of *Rps6* in domesticated and wild barley.

Map-based cloning of *Rps6* will open up the possibility of transferring a nonhost resistance gene into the host species, wheat. Whether *Rps6* would retain functionality in wheat is unclear and would depend upon the species conservation of the mechanisms underlying immunity. Wheat and barley diverged from a common ancestor approximately 11.6 million years ago (Wicker et al. 2009). Encouragingly, alleles of *Mla* from barley retained functionality when transferred to an immuno-compromised *Arabidopsis thaliana* accession (Maekawa et al. 2012). This demonstrated conservation of the underlying immune systems in two species that evolutionarily separated ~200 million ago. Cloning and intergenera transfer of *Rps6* and other genes contributing to the intermediate host status of barley will establish if barley may be used as a resource for the improvement of wheat.

### Author contribution statement

Conceived and designed the experiments: AMD JNF MJM. Performed the experiments: AMD JNF MG PG AH MJM. Analyzed the data: AMD JNF MJM. Wrote the paper: AMD MJM.

## Electronic supplementary material

Below is the link to the electronic supplementary material.

CAPS markers developed in the *Rps6* region (XLSX 50 kb)

Sequence used for Sequenom marker development in the *Rps6* region (XLSX 64 kb)

KASP markers developed in the *Rps6* region (XLSX 48 kb)

Histogram of macroscopic phenotypes of the Abed Binder 12 x Russell F_2_ population inoculated with *Psh* isolate B01/2. Parental and F_1_ phenotypes shown above plots (A: Abed Binder 12, R: Russell). (TIFF 1052 kb)

Genetic map of the Abed Binder 12 x Russell F_2_ population using 362 non-redundant markers. Genetic distances were calculated using the Kosambi map function in cM (TIFF 1955 kb)

Two-point linkage test of the Abed Binder 12 x Russell F_2_ population genetic map (TIFF 4310 kb)

Isolation of *Rps6* using genotypic and phenotypic selection. (a) and (b) show phenotype by genotype plots for chlorosis and pCOL phenotypes, respectively, for the Abed Binder 12 x Russell F_2_ population inoculated with *Pst* isolate 08/501. (c) and (d) show phenotype by genotype plots for chlorosis and pCOL phenotypes, respectively, for the single Abed Binder 12 x Russell F_2:3_ family inoculated with *Pst* isolate 08/21 (TIFF 1026 kb)

Uncoupling of *Psh* and *Pst* resistance in Abed Binder 12. (a) Phenotype by genotype plot using the chlorosis phenotype from the Abed Binder 12 x Russell F_2_ population inoculated with *Pst* isolate 08/501 at marker 2_0962. (b) Phenotype by genotype plot using the McNeal phenotype from the Abed Binder 12 x Russell F_2_ population inoculated with *Psh* isolate B01/2 at marker 2_0962 (TIFF 778 kb)

ANOVA analysis of *Rps6* and the chromosome 3H QTL in the Abed Binder 12 x Russell F_2:3_ population inoculated with *Pst* isolate 08/21 (XLSX 49 kb)
