# Supplementary figures and images for: Isolation and fine mapping of Rps6: an intermediate host resistance gene in barley to wheat stripe rust
Source: Theor Appl Genet. 2016 Jan 11;129:831–43. doi: 10.1007/s00122-015-2659-x (PMC4799244; doi:10.1007/s00122-015-2659-x)

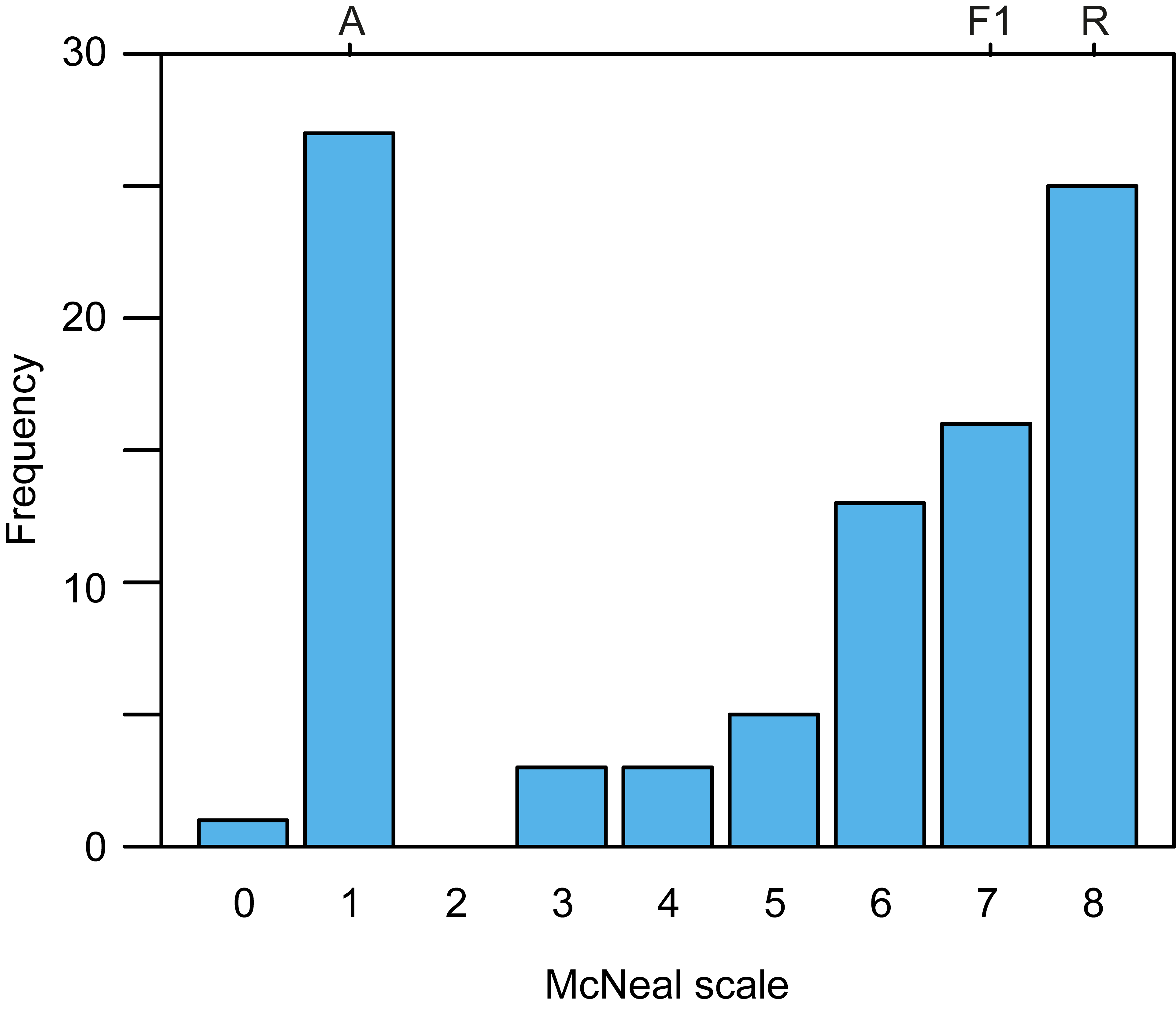

Supplement: Supplementary file 4 — Histogram of macroscopic phenotypes of the Abed Binder 12 x Russell F2 population inoculated with Psh isolate B01/2. Parental and F1 phenotypes shown above plots (A: Abed Binder 12, R: Russell). (TIFF 1052 kb) [file 122_2015_2659_MOESM4_ESM.tif]

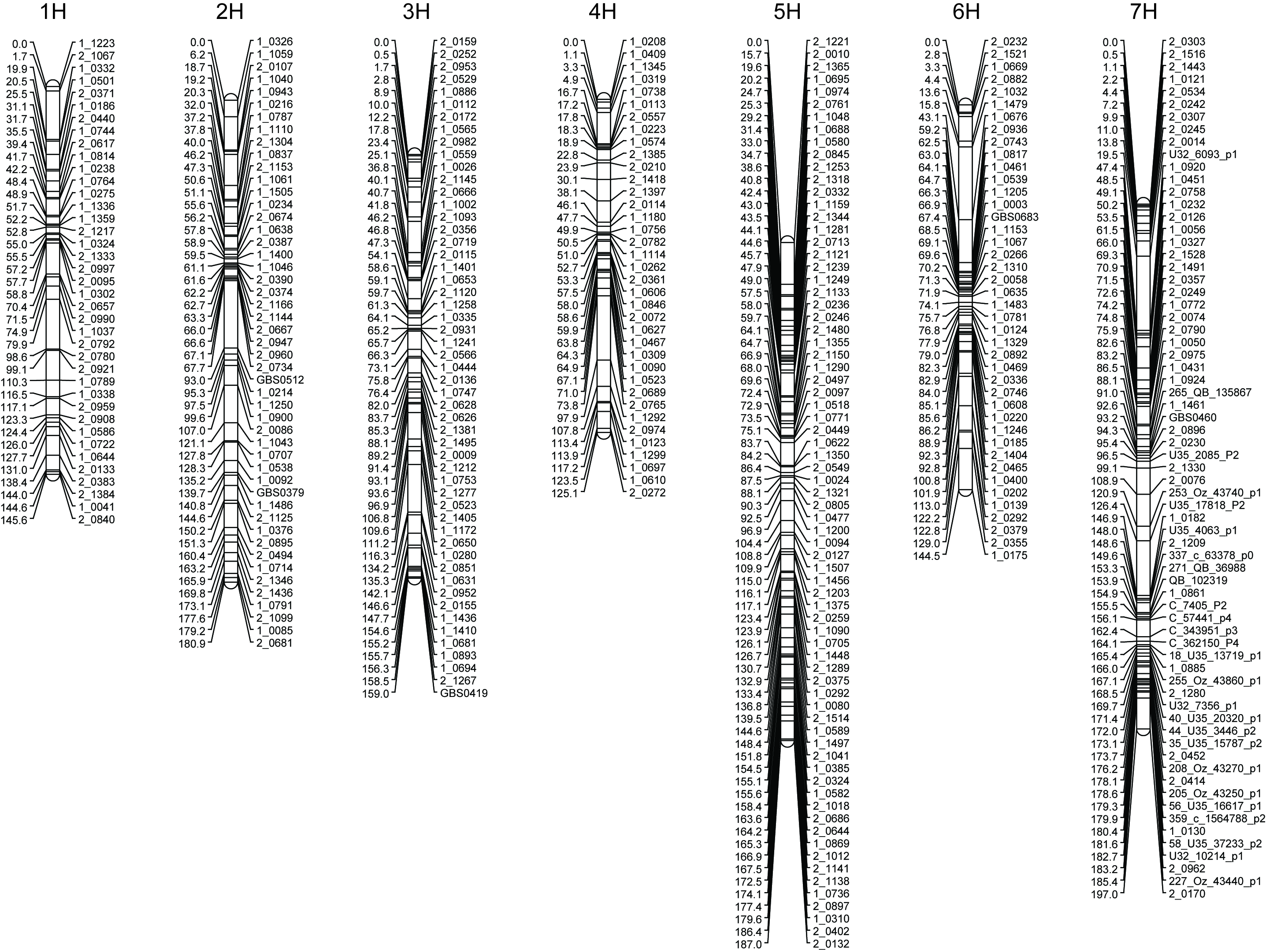

Supplement: Supplementary file 5 — Genetic map of the Abed Binder 12 x Russell F2 population using 362 non-redundant markers. Genetic distances were calculated using the Kosambi map function in cM (TIFF 1955 kb) [file 122_2015_2659_MOESM5_ESM.tif]

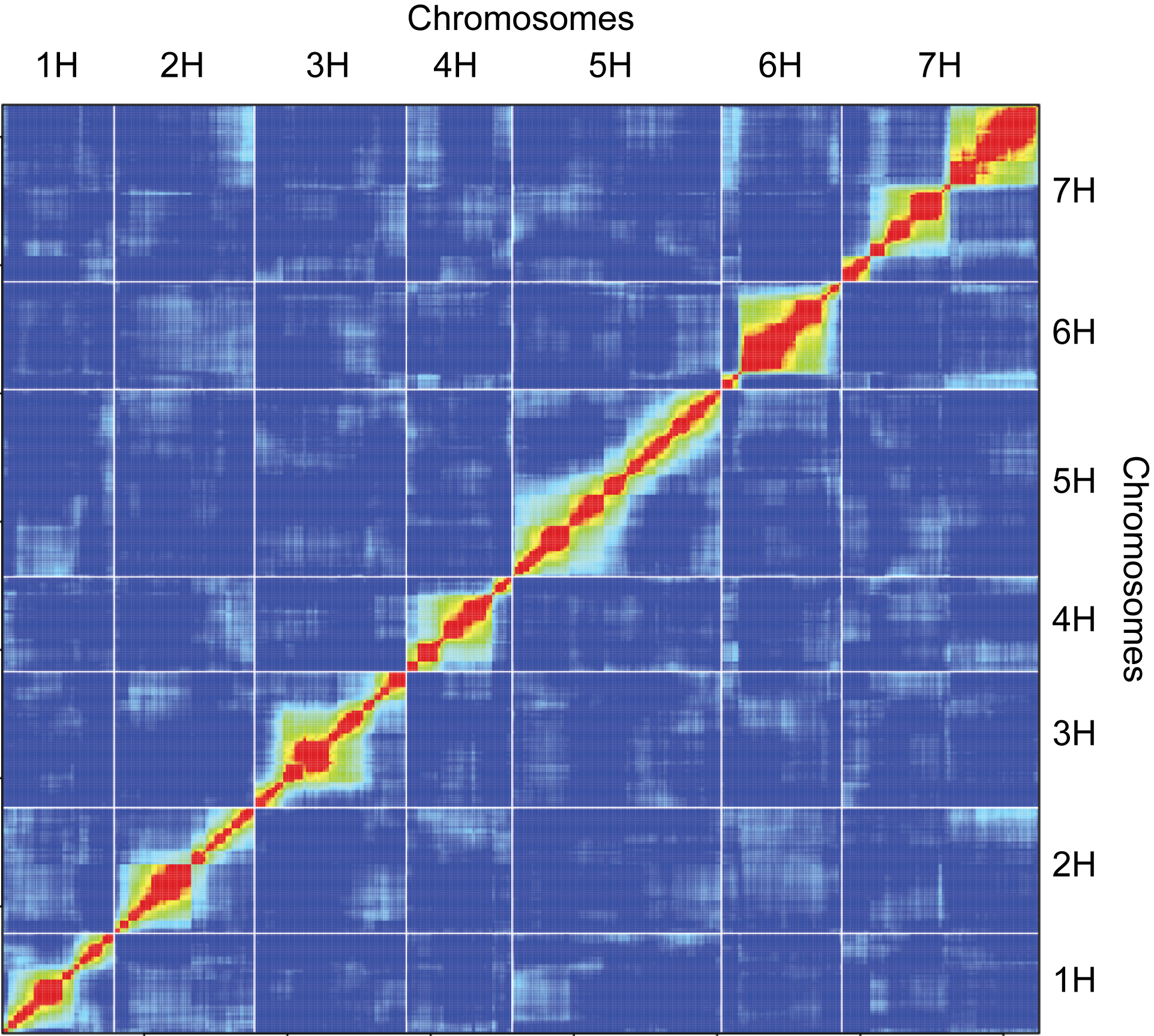

Supplement: Supplementary file 6 — Two-point linkage test of the Abed Binder 12 x Russell F2 population genetic map (TIFF 4310 kb) [file 122_2015_2659_MOESM6_ESM.tif]

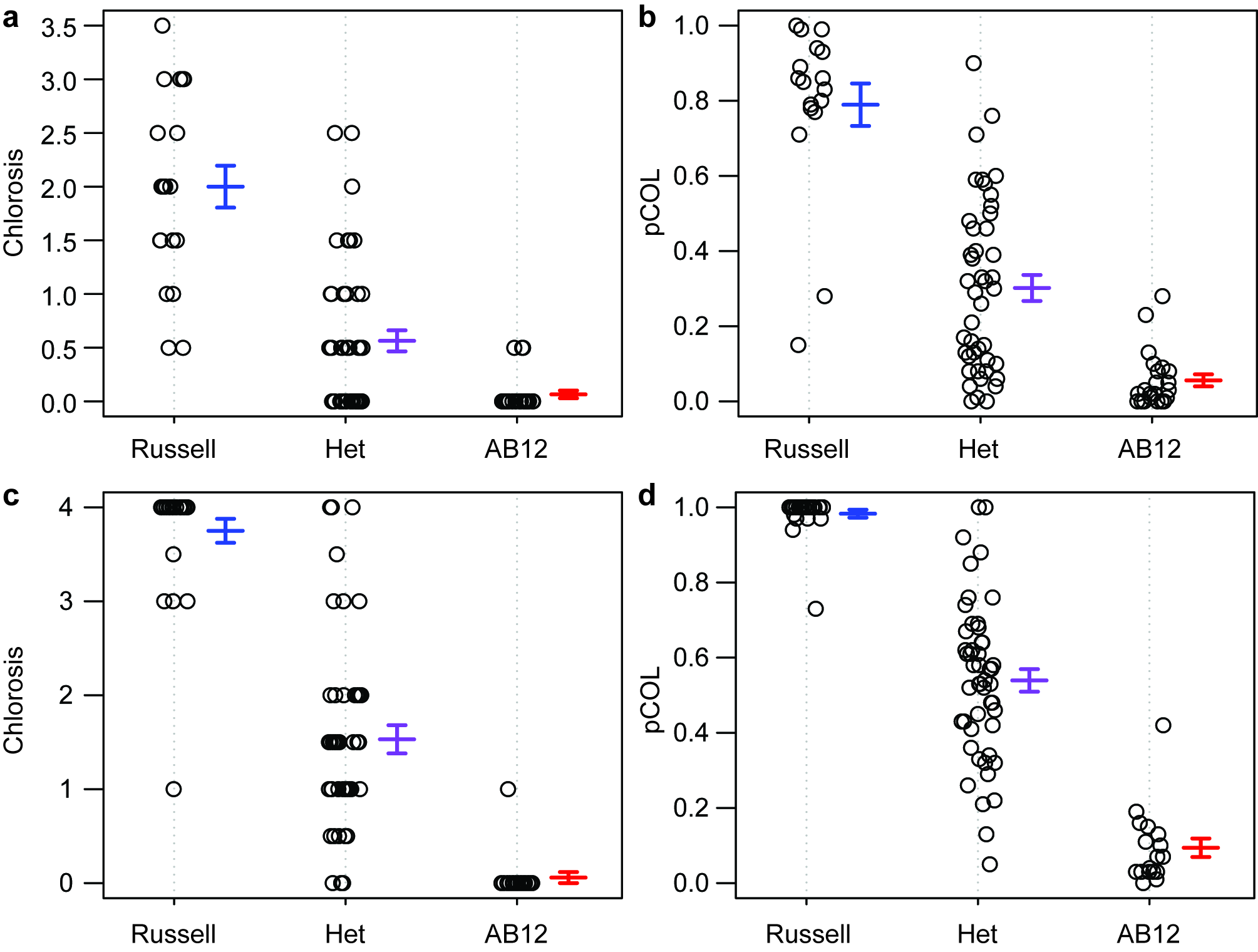

Supplement: Supplementary file 7 — Isolation of Rps6 using genotypic and phenotypic selection. (a) and (b) show phenotype by genotype plots for chlorosis and pCOL phenotypes, respectively, for the Abed Binder 12 x Russell F2 population inoculated with Pst isolate 08/501. (c) and (d) show phenotype by genotype plots for chlorosis and pCOL phenotypes, respectively, for the single Abed Binder 12 x Russell F2:3 family inoculated with Pst isolate 08/21 (TIFF 1026 kb) [file 122_2015_2659_MOESM7_ESM.tif]

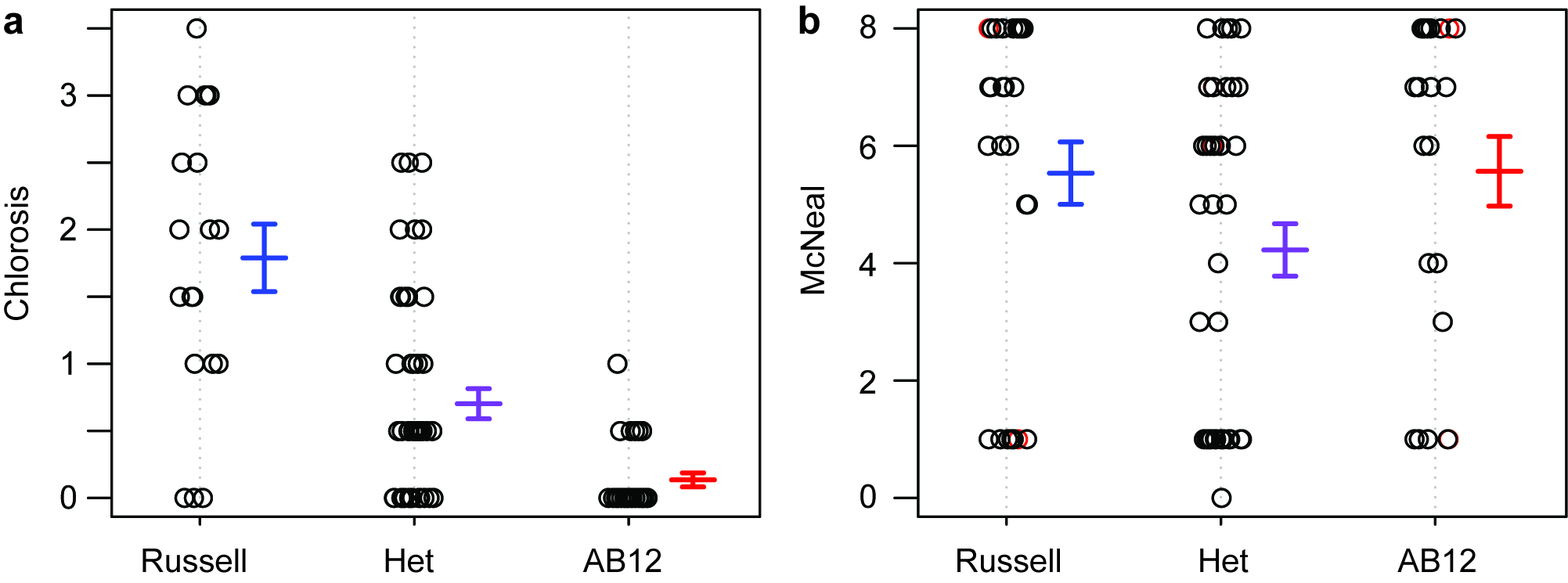

Supplement: Supplementary file 8 — Uncoupling of Psh and Pst resistance in Abed Binder 12. (a) Phenotype by genotype plot using the chlorosis phenotype from the Abed Binder 12 x Russell F2 population inoculated with Pst isolate 08/501 at marker 2_0962. (b) Phenotype by genotype plot using the McNeal phenotype from the Abed Binder 12 x Russell F2 population inoculated with Psh isolate B01/2 at marker 2_0962 (TIFF 778 kb) [file 122_2015_2659_MOESM8_ESM.tif]
